# A new insight of structures, bonding and electronic properties for 6-mercaptopurine and Ag_8_ clusters configurations: a theoretical perspective

**DOI:** 10.1186/s13065-019-0573-z

**Published:** 2019-04-19

**Authors:** Hongjiang Ren, Fan Chen, Xiaojun Li, Yaping He

**Affiliations:** Key Laboratory for Surface Engineering and Remanufacturing in Shaanxi Province, School of Chemical Engineering, Xi’an University, Taibai South Road 168#, Xi’an, 710065 China

**Keywords:** 6-Mercaptopurine, Ag_8_ cluster, Density functional theory, Density of states

## Abstract

**Background:**

Many reports have also shown that the silver nanoparticles can effectively increase the anticancer drug activity and intensely enhance the drug curative effect. The adsorption of 6MP on nanomaterials has received a lot of attentions because of the drug coordination to its chemotherapeutic activity. The geometrical structures, chemical bonds, molecular orbital properties as well as density of states for the configurations were analyzed to deeply understand the interactions between the 6MP and Ag_8_ clusters for high effect anticancer drug production.

**Results:**

In this work, the density functional theory B3LYP has been used to investigate the structures and properties of the configurations between 6-mercaptopurine (6MP) and Ag_8_ clusters using 6-311++G** level as well as an effective pseudo potential LANL2DZ. The geometries of ten configurations were optimized with full freedom. The geometrical structures, chemical bonds, molecular orbital properties as well as density of states for partial configurations were analyzed based on the density functional calculations. Polarizable continuum solvent model (PCM) in self-consistent reaction field (SCRF) were used for the aqueous calculations. The influences of temperature and pressure on the stability of the predominant configurations in the gas phase were further considered using standard statistical thermodynamic methods from 50 to 500 K and at 1 bar or 100 bar.

**Conclusion:**

The result shows that there are ten stable configurations in the gas phase and there is a strong chemical bond between a Ag and S atom in the most stable configuration. The analysis of density of states also shows that the Ag–S chemical bond in the most stable configuration has been formed. Moreover, the results show that the temperature and the pressure will significantly influence the stability of the configurations in the gas phase. Additionally, when the solvent effect was considered, we found that there are only seven stable configurations and the solvent have different effect on various configurations.
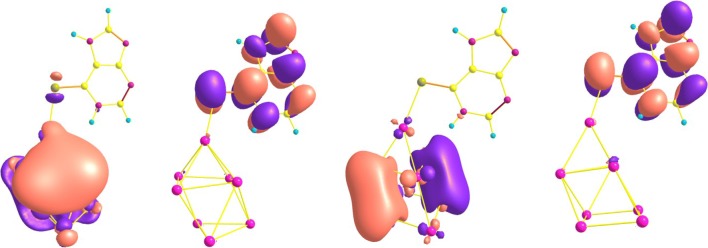

**Electronic supplementary material:**

The online version of this article (10.1186/s13065-019-0573-z) contains supplementary material, which is available to authorized users.

## Introduction

Nanotechnology is swiftly distensible in many fields including synthesis, catalysts, separation, chemiluminescence and sensing [1, 2]. Because of their unique physical and chemical properties such as large surface area, sensitivity and activity, the nanomaterials and products were required for many chemical reactions including the enhancers, catalysts, absorbing materials, energy acceptors and so on [3, 4]. Silver nanoparticles (AgNPs) are easy to be composed from inexpensive forerunners, which have displayed many optical characteristics and possessed good chemical stability, dramatic catalytic property and electrocatalytic property. It has also been found that the silver nanoparticles can effectively increase the anticancer drug activity and intensely enhance the drug curative effect [5–9]. At present, many first-principle reports have been limited to the Ag_n_ clusters with n ≤ 13 and many candidate structures were considered [10, 11]. Huda and co-workers [10] have investigated the electronic and geometric structures of neutral, cationic, and anionic Ag_n_ (n = 5–9) clusters using second-order many-body perturbation theory (MP2), which shows that the Ag_8_ cluster is a magic-number cluster with the highest binding energy, a relative higher adiabatic and vertical ionization potential, and a lower electron affinity among all the considered clusters. Fernandez and co-workers [11] systematically studied the geometrical structure and electronic properties of neutral, cationic and anionic metal clusters M_n_ (M=Cu, Ag or Au, n = 2–13). Recently it is shown that Ag_8_ neutral clusters embedded in an argon matrix have a strong fluorescence signal [12].

6-Mercaptopurine (6MP) has been utilized as the chemotherapy drug for many years. It is widely used as an effect medicine in treating a variety of diseases, such as rheumatologic disorder, lymphoblastic leukaemia, inflammatory bowel disease and prevention of rejection following organ transplantation [13–22]. The adsorption of 6MP on nanomaterials has received a lot of attentions because of the drug coordination to its chemotherapeutic activity [23–25]. Vivoni and co-workers [24] have experimentally reported the interaction between 6MP and a silver electrode using surface-enhanced Raman spectroscopy (SERS) and Urey-Bradley force field combined with semiempirical PM3 calculation. It was concluded that when the 6MP molecule is adsorbed onto a silver electrode surface, it can attach via the N(1) atom. Chu and co-workers [25] have carried out self-assembled monolayers (SAMs) research of 6-mercaptopurine (6MP) on a silver electrode in acid and alkaline medias via the SERS technique combined with Raman mapping. It was found that the adsorption mode of 6MP would change with the pH data of the environment. The coordination of 6MP onto nanomaterials has been investigated, which can lead to slow releasing drugs of 6MP [26] and exploiting the reaction for heavy-metal determination [27]. Although there are many investigations about the coordination of 6MP to Ag nanoparticles forming AgNPs [10, 11, 23–27], they are only limited to investigate the experimental Raman spectroscopy and the scales and so on. What are the adsorption structures? What are the bonding properties? How can the temperature and pressure affect the adsorption of 6MP onto the Ag clusters? These problems have been not been answered till today.

In this work, we will provide the answers to the former problems throughout carrying out the detailed density functional theory calculations using B3LYP/6-311++G** level. The stabilities of ten configurations between two 6MP tautomers and Ag_8_ clusters will be explored. The geometrical structures, chemical bonds, molecular orbital properties as well as density of states for partial configurations were analyzed to understand the interactions between the 6MP and Ag_8_ clusters.

## Methods

In this work, the theoretical calculations were performed using the Gaussian 09 program package [28]. Density-functional theory (DFT) has also been employed in conjunction with pseudopotentials to study silver clusters. The geometries of all the configurations were optimized with DFT-B3LYP method combined with 6-311++G** basis set for non-metal atoms (C, H, N, S) and an effective pseudo potential basis set LANL2DZ only for Ag atoms. In this core potential, the inner 28 electrons (1s^2^2s^2^2p^6^3s^2^3p^6^3d^10^) are replaced by RECP and the outer 19 electrons (4s^2^4p^6^4d^10^5s^1^) are taken as valence electrons. The optimizations were performed in all degrees of freedom. Harmonic vibrational frequencies were analyzed to verify the stationary points at the same theoretical level. Zero-point vibrational energies (ZPVE) and thermal corrections were evaluated under standard state conditions (298.15 K and 100 kPa) via frequency analysis. The thermochemistry energy parameters were calculated at the B3LYP functional level of theory. The binding energies between the Ag_8_ clusters and 6MP molecule were denoted by Δ*E*_b_, which were given for per configuration:

1 $$\Delta E_{\text{b}} = \, - \, \left[ {E\left( {{\mathbf{Cn}}} \right) \, - E\left( {{\mathbf{Ag}}_{{\mathbf{8}}} } \right) \, - E\left( {{\mathbf{6MP}}{ - }{\mathbf{7}}} \right)} \right]$$

Here is an example for **6MP-7**, in which n represents the labeled number. For the configurations, it is named as **Cn** (n = 1–10). For the **6MP-9**, it is the same method with **6MP-7**, in which, **6MP-7** and **6MP-9** are the two isolated tautomers. In the aqueous calculations, the solvent effect (water) has been considered using continuum models: polarizable continuum model (PCM) in self-consistent reaction field (SCRF). As usual, the solvent is viewed as a continuous dielectric medium characterized by a uniform dielectric constant (ε) in this model (ε = 78.39 for water).

The bonding properties and bond strength variations were analyzed based on the NBO calculations using the NBO package version 3.1, which were conducted at the DFT-B3LYP/6-311++G**//LANL2DZ theoretical level, implemented in the Gaussian 09 program. The analysis can provide a comprehensive insight for the configurations of 6-mercaptopurine (6MP) on Ag_8_ clusters.

Heat capacity, entropy, enthalpy and Gibbs free energy of the corresponding four configurations at 298.15 K were evaluated with the standard statistical thermodynamics procedures by using the B3LYP/6-311++G** geometries and frequencies. The heat capacities as a function of temperature in 50–500 K were also developed with the same method and fitted into an analytical equation (Eq. 2). By using the heat capacities, the energy at any temperature (e.g., 500 K) were derived classically.

2 $$C_{p,m}^{\theta } = a_{0} + a_{1} T + a_{2} T^{2} + a_{3} T^{3} + a_{4} T^{ - 2}$$

## Results and discussion

### Structures and energies

**Fig. 1:**
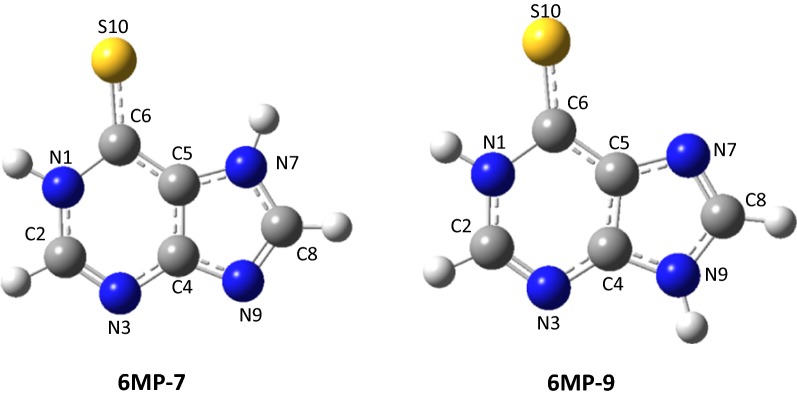
Geometries and atomic number of two tautomers for 6-mercaptopurine (6MP)

**Fig. 2:**
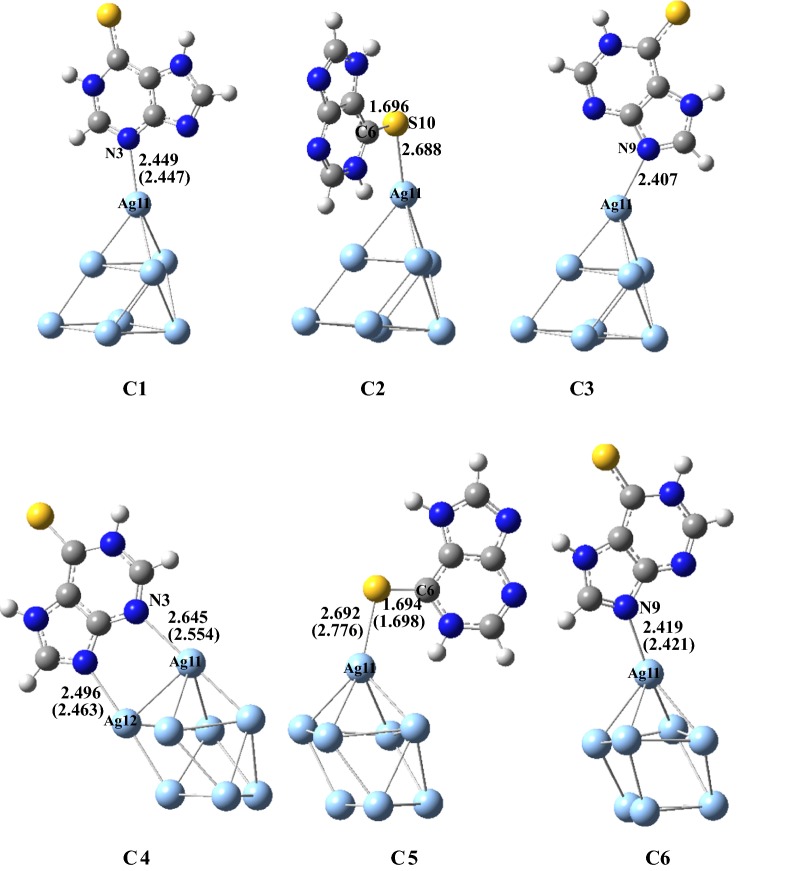
Six geometries of the formed configurations on **6MP-7** with the Ag_8_ clusters (Bond lengths are given in Å and the data in the parentheses is in the aqueous phase)

**Fig. 3:**
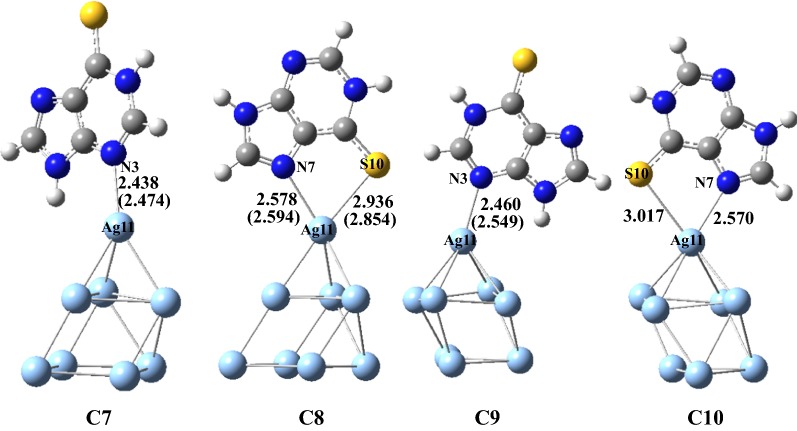
Four geometries of the formed configurations on **6-MP-9** with the Ag_8_ clusters (Bond lengths are given in Å and the data in the parentheses is in the aqueous phase)

From Refs. [29, 30], it can be seen that 6MP molecule can exist in the eight potential configurations, and the two tautomers **6MP-7** and **6MP-9** can be predominant with the large quantity in the gas and aqueous phases. Other six tautomers can exist in a little quantity. Hence, in this work, the two tautomers **6MP-7** and **6MP-9** are considered for the next discussions and the geometries as well as atomic number of two tautomers **6MP-7** and **6MP-9** were shown in Fig. 1. It can be seen from Fig. 1 that when considering possible coordination atoms, three potential positions N(3), N(7) and S(10) for **6MP-7** or N(3), N(9) and S(10) for the molecule **6MP-9** can be attacked by the metal Ag atom, mainly because of the lone pair electrons for the N and S atoms being extremely important for promoter action. Therefore when considering the adsorption reaction, the orientation of adsorbed **6MP** is critical in its action as a mediator of electron transfer. According to the reports by Huda et al. [10], the Ag_8_ cluster is a magic-number cluster among the investigated clusters and it has two representative superiority structures. And two potential configurations were considered in this work based on the reported structures [10]. Six stable configurations of **6MP-7** adsorbed onto the Ag_8_ clusters surface were shown in Fig. 2, which are named as **C1**, **C2**, **C3**, **C4**, **C5** and **C6**. Four stable ones of the **6MP-9** with the Ag_8_ clusters were also given in Fig. 3, which are named as **C7**, **C8**, **C9** and **C10**. Additional file 1: Table S1 gives out the zero point energy, total energy, standard Gibbs free energy and enthalpy of all the configurations at the temperature 298.15 K with B3LYP/6-311++G**//LANL2DZ dual level. The relative energy data is shown in Table 1.

**Table 1:** Relative standard Gibbs free energy (*∆*G^θ^, kJ/mol) at 298.15 K obtained at the B3LYP/6-311++G**//LANL2DZ level for all the complexes both in the gas and aqueous phases

Complexes	In the gas phase ∆G^θ^	In the aqueous phase ∆G^θ^
**6MP-7**		
**C1**	10.41	8.35
**C2**	1.77	–
**C3**	7.17	–
**C4**	11.73	16.14
**C5**	0.00	0.00
**C6**	15.08	9.03
**6MP-9**		
**C7**	27.69	14.19
**C8**	9.46	9.75
**C9**	26.09	9.93
**C10**	12.11	–
**Reactants**		
**6MP-7 + Ag**_**8**_**(1)**^a^	3.59	− 10.67
**6MP-7 + Ag**_**8**_**(2)**^b^	2.72	–
**6MP-9 + Ag**_**8**_**(1)**	15.77	− 7.68
**6MP-9 + Ag**_**8**_**(2)**	14.90	–

^a^**Ag**_**8**_**(1)** represent the a trigonal pyramid Ag_8_ cluster over an approximate square

^b^**Ag**_**8**_**(2)** represent the a square pyramid Ag_8_ cluster over a triangle. But in the aqueous phase it is not found

It is seen from Figs. 2, 3 that the structure of Ag_8_ cluster in the configurations **C1**, **C2**, **C3**, **C7** and **C8** is dramatically different from that of **C4**, **C5**, **C6**, **C9** and **C10**. In the configurations **C1**, **C2**, **C3**, **C7** and **C8**, the Ag_8_ cluster owned a trigonal pyramid over an approximate square and however, in **C4**, **C5**, **C6**, **C9** and **C10** the Ag_8_ cluster owned a square pyramid over a triangle. In the two clusters configurations, the purine ligand attached to the top vertex of the corresponding pyramid. In the **C4** configuration, there is forming a two center coordination of the ligand to the two Ag atoms of the metal cluster. In the **C8** and **C10** configurations, there are forming a bidentate coordination of the ligand to only one Ag atom of the metal cluster. Other configurations formed a single coordination between the ligand molecule and Ag_8_ clusters. From Table 1, the lowest energy configuration is **C5** and the energy was taken as a zero. And the relative energies of other nine configurations are based on this. And the second one is **C2** with a relative energy of 1.77 kJ/mol. The third and fourth ones are **C3** and **C8** with a relative energy of 7.17 and 9.46 kJ/mol, respectively. The relative Gibbs free energies of other six configurations **C1**, **C4**, **C10**, **C6**, **C9** and **C7** are 10.41, 11.73, 12.11, 15.08, 26.09 and 27.69, respectively. These results show that the configuration **C5** is the most stable one of all the configurations and **C2** is also possibly predominant one. The configurations **C3** and **C8** can exist in large quantity because of lower relatively energies. Other configurations have slightly higher energies and the stabilities are slightly lower than that of the former four configurations. When these configurations were put into the water solvent, we found that the **C2**, **C3** and **C10** configurations can not been obtained. Even now, in the gas phase discussion, we still consider **C2** and **C3** for comparisons with other configuration.

In the configuration **C5**, the Ag11–S10 bond is formed with a distance of 2.692 Å and is slightly longer than the experimental data of 2.48 Å [18], implying that there is existing the classical chemical bonding between the Ag(11) atom and S(10) atom. The chemical bond S(10)–C(6) has been lengthened to 1.694 Å from the 1.670 Å by 0.024 Å and the N(1)–H has also been lengthened to 1.025 Å from the 1.013 Å by 0.012 Å. In **C2**, the chemical bond S(10)–C(6) is 1.688 Å and in the configurations **C1**, **C3**, **C4** and **C6**, the N(3)(or N(9))–Ag(10) chemical bonds are 2.449, 2.407, 2.645 and 2.419 Å, respectively, suggesting that the Ag–S or Ag–N chemical bonds are formed in the end. In these six configurations, the different positions can be attacked by Ag atom of the Ag_8_ clusters. In **6MP-9** configurations, we try to find all the configurations and but only four possible configurations were found. All the structures are shown in Fig. 2. In these four configurations, the Ag atom also formed the chemical bond with the N or S atoms, and it can seen that in **C2** and **C4**, the N and S atoms simultaneously formed the chemical bonds with one Ag atom. In **C2**, the N(7)–Ag(11) and S(10)–Ag(11) bond lengths are 2.578 and 2.936 Å, respectively. And in **C4**, the N(7)–Ag(11) and S(10)–Ag(11) bond lengths are 2.570 and 3.017 Å, respectively. However, in **C1** and **C3**, only the single N(3)–Ag(11) bond is formed with the distances of 2.438 and 2.460 Å, respectively. These data show that there are existing strong chemical bonds between the 6MP and Ag_8_ clusters.

When these configurations were put into the water solvent, we found that the most largest data of bond length changing is Ag–N bond in **C4** and it is decreasing with 0.091 Å. The second is Ag–N bond in **C9** and it is increasing with 0.089 Å. In **C5**, the Ag–S bond is also increasing with 0.084 Å and in C8, the Ag–S bond is also decreasing with 0.082 Å. Other bonds are changing slightly. Obviously, the solvent have different effect on various configurations.

### Binding energies

Table 2 gives out the binding energies of ten configurations, in which, Δ*E*_*b*_ is the uncorrected interaction energy without basis set superposition error (BSSE) and Δ*E*_*b*_ (BSSE) is the corrected interaction energies including BSSE corrections. In Table 2, it can be concluded that BSSE data are 5.27–6.95 kJ/mol, which are 10.46–17.06% of the uncorrected interaction energies, suggesting that the basis set effect should be included in this work. In the next discussion, the corrected energies are used for illustrating these problem. In Table 2, the obtained binding energy of **C8** is 50.63 kJ/mol and that of **C2** is 48.70 kJ/mol. And the binding energy difference between them is 1.93 kJ/mol. The obtained binding energies of **C5** and **C10** are 46.61 and 44.02 kJ/mol, respectively. The binding energies of **C1**, **C3**, **C4**, **C6**, **C7** and **C9** are 33.72, 39.12, 36.44, 35.61, 30.70 and 28.95 kJ/mol, respectively and the average binding energy of these six configurations are 34.09 kJ/mol. The result illustrates that the interaction effects between **6MP** and Ag_8_ clusters in **C8** and **C2** are larger. From Table 2, it can be seen that the lower relative Gibbs free energies, the larger the binding energies of those configurations especially for the configurations **C2**, **C5** and **C8**.

**Table 2:** Binding energy (Δ*E*_*b*_) and corrected binding energy (Δ*E*_*b*_) including BSSE of all the complexes (kJ/mol) in the gas phase

Complexes	Δ*E*_*b*_	Δ*E*_*b*_ (BSSE)	Δ_*BSSE*_	Complexes	Δ*E*_*b*_	Δ*E*_*b*_ (BSSE)	Δ_*BSSE*_
**C1**	39.00	33.72	5.28	**C7**	36.65	30.71	5.94
**C2**	54.98	48.70	6.28	**C8**	56.53	50.63	5.90
**C3**	44.39	39.12	5.27	**C9**	34.90	28.95	5.95
**C4**	43.39	36.44	6.95	**C10**	50.08	44.02	6.06
**C5**	52.76	46.61	6.15				
**C6**	41.00	35.61	5.39				

However, the binding energy of **C3** is lower than **C2**, which can attribute to the various interaction between **6MP** and Ag_8_ cluster. Seen from Fig. 3, in **C8** there are forming two chemical bonds for N(7)–Ag(11) and S(10)–Ag(11), which results from the double coordination of **6MP** and Ag_8_. And the interaction effect should be much stronger than that of single coordination of Ag_8_ and **6MP** in **C3** or **C5**, which can explain why the configuration **C8** has the greatest binding energy. In order to further expose the bonding property between the **6-MP** molecules and Ag_8_ clusters, the following orbital analysis are carried out for answering the problems.

### Orbital analysis

**Fig. 4:**
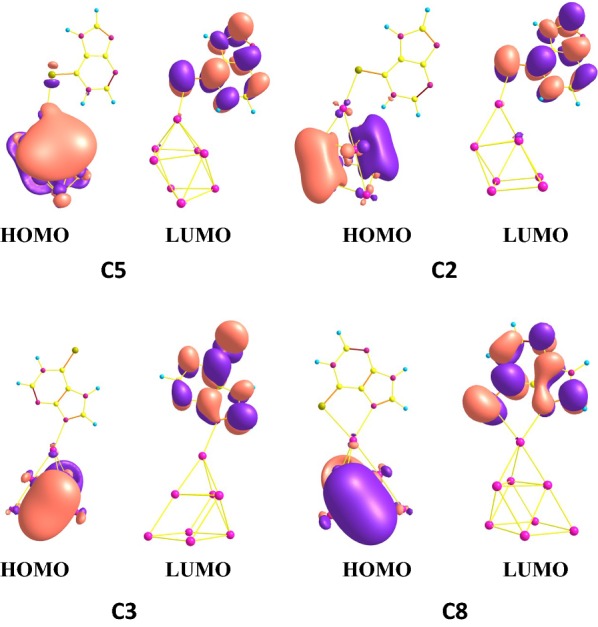
Frontier molecular orbits (HOMO and LUMO) of the considered four configurations **C5**, **C2**, **C3** and **C8**

The highest occupied molecular orbital (HOMO) and lowest unoccupied molecular orbital (LUMO) of the four relative stable configurations **C2**, **C3**, **C5** and **C8** are depicted in Fig. 4. It is shown that the HOMO orbital electrons of **C5** are mainly delocalized in the Ag_8_ clusters, which are far from the **6MP** molecule. The contributions could not be found from the purine ring, suggesting that the HOMO contribution does not mainly result from the purine ring. However, the LUMO electrons are delocalized in the purine ring and S atom via delocalized π bond. These contributions mainly attributed to the covalent bond in the purine ring. The HOMO and LUMO distributions illustrate that **C5** has the largest energy gap. The electronic clouds of the HOMO orbital in **C2**, **C3** and **C8** configurations are not much larger than that of **C5** because of little overlapping field of Ag d electrons, but the LUMO electronic mainly attributed to anti-bonding orbital of purine ring, showing that these configurations have smaller gaps. At the same time, the energy gaps of four configurations **C2**, **C3**, **C5** and **C8** are 198.83, 159.50, 221.75 and 146.03 kJ/mol, showing that among these configurations, **C5** has the highest one. And moreover, the stability for **C5** is also the largest one of the four configurations, which is almost same with the predicted stability from energy information. For further exposing the bonding properties, the NBO analysis obtained at B3LYP/6-311++G**//LANL2DZ level of theory are listed in Table 3. Seen from Table 3, the second order perturbation interaction energy LP[S(10)] → LP*[Ag(1)] of **C5** is 101.25 kJ/mol and the LP[S(10)] → LP*[Ag(1)] interaction of **C2** is 92.76 kJ/mol, implying that there are existing strong chemical bonds and the interaction energies of **C2** and **C8** are 57.66 and 50.38 kJ/mol, respectively. As we can know, the second order perturbation interaction energy can show the strength of formed chemical bonds.

**Table 3:** Second order interaction energies in NBO analysis at B3LYP/6-311++G** level

Complexes	Donor → acceptor	*E*^(2)^/(kJ/mol)
C5	LP[S(10)] → LP*[Ag(11)]	101.25
C2	CR[S(10)] → LP*[Ag(11)]	25.98
	LP[S(10)] → LP*[Ag(11)]	92.76
	LP[Ag(1)] → BD*[S(10)–Ag(11)]	13.14
C3	LP[N(9)] → LP*[Ag(11)]	57.66
C8	LP[S(10)] → LP*[Ag(11)]	50.38

### Density of states analysis

**Fig. 5:**
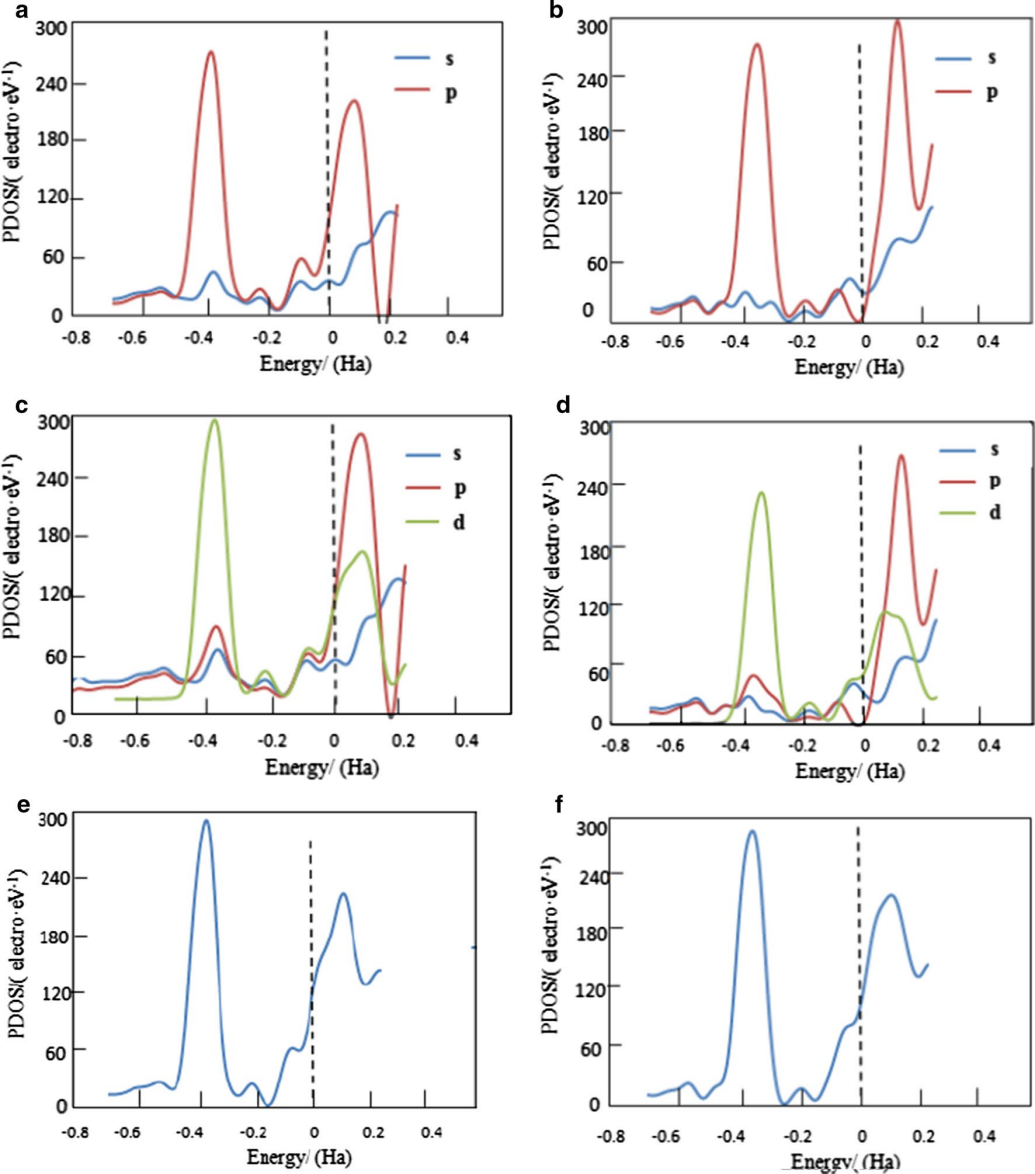
Densities of states of **C5** (**a**, **c**, **e**) and **C8** (**b**, **d**, **f**) for the most stable two adsorption configuration. **a**, **b** Represent the electron density of molecule after adsorption; **c**, **d** Represent electron density of Ag surface after adsorption; **e**, **f** represent total DOS most stable adsorption configuration

In order to well understand the electronic properties of the titled clusters and the interaction between Ag_8_ clusters and **6MP** molecule, the density of states of two more stable adsorption configurations **C5** and **C8** were calculated, as shown in Fig. 5, because the energies of **C5** and **C8** in the solvent are relatively lower than others. In Fig. 5a, b represent the electron density of molecule after adsorption (c, d) represent electron density of Ag surface after adsorption (e, f) represent total DOS most stable adsorption configuration. In the present work, the density of states near fermi levels were focused because those density of states near the Fermi levels are significant. As seen from the Fig. 5, it is shown that for the density of states near the Fermi levels, the contributions of p electrons are the maximum and the contributions of s electrons are the minimum for the **6MP** molecule after adsorption shown in (a, b), and that of d electrons are in between p electrons and s electrons for **C8** from (d) and that of d electrons are the maximum for **C5** from (c). Also seen from Fig. 5, in the negative field, there are strong interaction between the p electrons of molecules and d electrons of Ag clusters. Hence, the p electrons have the largest contributions to the Fermi levels, which may be originated from that the Ag elements are the evident p-block elements, which have resulted from the **6MP** molecules adsorption on Ag_8_ clusters. For the density of states in the range of − 0.5 to − 0.3 Ha, the p electrons almost have the same contributions from (a, b), whereas s electrons have the smallest contributions. In comparison with (c, d), it also shows that d contribution from **Ag**_**8**_ in **C5** are more than that in **C8**, which is in agreement with the most stable order of the configuration **C5**.

### Temperature and pressure effect

**Fig. 6:**
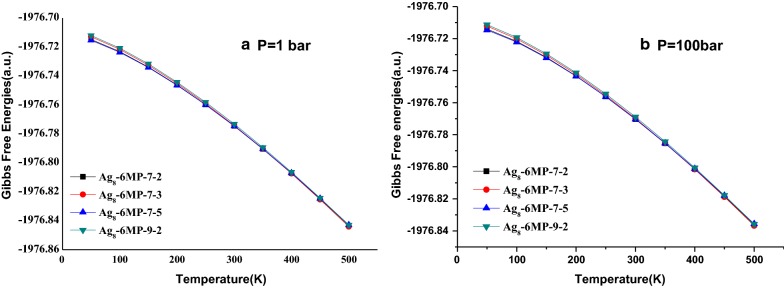
The Gibbs free energies of the considered configurations **C5**, **C2**, **C3** and **C8** with the temperature increasing from 50 to 500 K and pressures of 1 bar for (**a**) and 100 bar for (**b**)

For exploring the temperature and pressure influence on the configurations, the different temperatures and pressure are considered in this work. The temperature was from 50 to 500 K and the pressure was given at two conditions 1 bar and 100 bar. The Gibbs free energies obtained using the standard statistical thermodynamic method ranged from 50 to 500 K are employed in the next discussion. The relations between the Gibbs free energies and temperature for the configurations **C2**, **C3**, **C5** and **C8** at 1 bar and 100 bar are illustrated in Fig. 6a, b, respectively. As can be seen from Fig. 6a, b, the Gibbs free energies of the four configurations dramatically decrease with the increasing of the temperature from 50 to 500 K, implying that the temperature can affect the stability of the four configurations **C2**, **C3**, **C5** and **C8**. In Fig. 6, the Gibbs free energies differences of the four configurations are very slight regardless of at 1 bar or 100 bar, implying that the temperature can almost have same effect on the stabilities of the four configurations. Seen from Fig. 6 the Gibbs free energy of **C8** is the most highest among four configurations between 50 and 300 K either at 1 bar or 100 bar. The stabilities of **C2** and **C5** are also same within the investigated pressure and temperature range. The results also show that in different temperatures these configurations have different stabilities, which can be attributed to formed different chemical bonds. The stabilities of **C2** and **C5** are obviously lower than other two configurations.

## Conclusions

Theoretical investigations have been carried out for the configurations between 6-mercaptopurine and Ag_8_ cluster using the density functional method B3LYP with 6-311++G**//LANL2DZ basis set. The configurations geometrical structures were optimized with no constraints. The energies, bonding properties and density of states of the corresponding configurations were analyzed in detail. Additionally, the influences of temperature and pressure on the stability of the four configurations were also explored using standard statistical thermodynamic methods. The result shows that the configuration **C5** can be the most stable among the investigated configurations, in which the Ag(11) atom interacts with the S(10) atom forming the strong chemical bond. The density of states also show that the Ag–S chemical bond is formed and the d contribution from **Ag**_**8**_ in **C5** are more than that in **C8**, which support the most stable order of the configuration **C5**. The temperatures and the pressure can significantly influence the stability of the four stable configurations.

## Additional file

**Additional file 1: Table S1.** Zero point energy (*ZPE*, a.u.), total energy (*E*_T_, a.u.), standard Gibbs free energy (*G*^θ^, a.u.) and enthalpy (*H*^θ^, a.u.) at 298.15 K obtained at the B3LYP/6-311++G**//LanL2DZ level for ten complexes.

## References

[1] Brechignac C, Houdy P, Lahmani M (2007). Nanomaterials and nanochemistry.

[2] Karimi-Maleh H, Biparva P, Hatami M (2013). A novel modified carbon paste electrode based on NiO/CNTs nanocomposite and (9,10-dihydro-9, 10-ethanoanthracene-11, 12-dicarboximido)-4-ethylbenzene-1, 2-diol as a mediator for simultaneous determination of cysteamine, nicotin amide adenine dinucleotide and folic acid. Biosens Bioelectron.

[3] Su Y, Xie Y, Hou X, Lv Y (2014). Recent advances in analytical applications of nanomaterials in liquid-phase chemiluminescence. Appl Spectrosc Rev.

[4] Biparva P, Abedirad SM, Kazemi SY (2014). ZnO nanoparticles as an oxidase mimic-mediated flow-injection chemiluminescence system for sensitive determination of carvedilol. Talanta.

[5] Bagatur’yants AA, Safonov AA, Stoll H, Werner HJ (1998). Ab initio relativistic pseudopotential study of small silver and gold sulfide clusters (M_2_S)_n_, n = 1 and 2. J Chem Phys.

[6] Eachus RS, Marchetti AP, Muenter AA (1999). The photophysics of silver halide imaging materials. Annu Rev Phys Chem.

[7] Koretsky GM, Knickelbein MB (1997). The reactions of silver clusters with ethylene and ethylene oxide: infrared and photoionization studies of Ag_n_(C_2_H_4_)_m_, Ag_n_(C_2_H_4_O)_m_ and their deuterated analogs. J Chem Phys.

[8] Tani T (1989). Physics of the photographic latent image. Phys Today.

[9] Sieber C, Buttet J, Harbich W, Felix C (2004). Isomer-specific spectroscopy of metal clusters trapped in a matrix Ag_9_. Phys Rev A.

[10] Huda MN, Ray AK (2003). Electronic structures and magic numbers of small silver clusters: a many-body perturbation-theoretic study. Phys Rev A.

[11] Fernandez EM, Soler JM, Garzon IL, Balbas LC (2004). Trends in the structure and bonding of noble metal clusters. Phys Rev B.

[12] Félix C, Sieber C, Harbich W, Buttet J, Rabin I, Schulze W, Ertl G (2001). Ag_8_ fluorescence in argon. Phys Rev Lett.

[13] Bouhnik Y, Lemann M, Mary JY, Scemama G, Taı R, Matuchansky C, Madiliani R, Rambaud JC (1996). Long-up of patients with Crohn’s disease treated with azathioprine or 6-mercaptopurine. Lancet.

[14] Estlin EJ (2001). Continuing therapy for childhood acute lymphoblastic leukaemia: clinical and cellular pharmacology of methotrexate, 6-mercaptopurine and 6-thioguanine. Cancer Treat Rev.

[15] Rajapakse RO, Korelitz BI, Zlatanic J, Baiocco PJ, Gleim GWAm (2000). Outcome of pregnancies when fathers are treated with 6-mercaptopurine for inflammatory bowel disease. J Gastroenterol.

[16] Mutinga M, Castells M, Horan R, Farraye FA (2000). Successful desensitization to 6-mercaptopurine in a patient with Crohn’s disease. Am J Gastroenterol.

[17] Chuan D, Ding LH, Wei YX, Li JF, Wei YL (2003). Spectroscopy behavior of 6-mercaptopurine, azathiopurine, and 8-azaguanine. Spectrochim Acta A.

[18] Chen ZG, Zhang GM, Chen X, Chen JH, Liu JB, Yuan HQ (2013). A fluorescence switch sensor for 6-mercaptopurine detection based on gold nanoparticles stabilized by biomacromolecule. Biosens Bioelectron.

[19] Hawwa AF, Millership JS, Collier PS, McElnay JC (2009). Development and validation of an HPLC method for the rapid and simultaneous determination of 6-mercaptopurine and four of its metabolites in plasma and red blood cells. J Pharm Biomed Anal.

[20] Keyvanfard M, Khosravi V, Karimi-Maleh H, Alizad K, Rezaei B (2013). Voltammetric determination of 6-mercaptopurine using a multiwall carbon nanotubes paste electrode in the presence of isoprenaline as a mediator. J Mol Liq.

[21] Sun H, Wang T, Liu X, Chen P (2013). A sensitive inhibition chemiluminescence method for the determination of 6-mercaptopurine in tablet and biological fluid using the reaction of luminol–Ag(III) complex in alkaline medium. J Lumin.

[22] Wang L, Ling B, Chen H, Liang A, Qian B, Fu J (2010). Flow injection chemiluminescence determination of 6-mercaptopurine based on a new system of potassium permanganate thioacetamide sodium hexametaphosphate. Luminescence.

[23] Fischer B, Dubler E, Meienberger M, Hegetschweiler K (1998). Molybdenum complexes of the anticancer drug 6-mercaptopurine. Inorg Chim Acta.

[24] Vivon A, Chen SP, Ejeh D, Hosten CM (2000). Determination of the orientation of 6-mercaptopurine adsorbed on a silver electrode by surface-enhanced raman spectroscopy and normal mode calculations. Langmuir.

[25] Chu H, Yang HF, Huan SY, Shen GL, Yu RQ (2006). Orientation of 6-mercaptopurine SAMs at the silver electrode as studied by raman mapping and in situ SERS. J Phys Chem B.

[26] Mondal BC, Das D, Das AK (2001). Application of a new resin functionalised with 6-mercaptopurine for mercury and silver determination in environmental samples by atomic absorption spectrometry. Anal Chim Acta.

[27] San B, Dela-Riva V, Costa-Fernandez JM, Pereiro R, Medel AS (2000). Fluorimetric method for the determination of trace levels of mercury in sea water using 6-mercaptopurine. Anal Chim Acta.

[28] Frisch MJ, Trucks GW, Schlegel HB, Scuseria GE, Robb MA, Cheeseman JR, Scalmani G, Barone V, Mennucci B, Petersson GA (2009). Gaussian 09, Revision D. 01.

[29] Civcir PU (2001). A theoretical study of tautomerism of 6-thiopurine in the gas and aqueous phases using AM1 and PM3. J Mol Struct THEOCHEM.

[30] Li BZ (2004). DFT calculations of 6-thiopurine tautomers. Acta Chim Sin.

