# Supplementary material for: A new insight of structures, bonding and electronic properties for 6-mercaptopurine and Ag8 clusters configurations: a theoretical perspective
Source: BMC Chem. 2019 Apr 19;13(1):55. doi: 10.1186/s13065-019-0573-z (PMC6661816; doi:10.1186/s13065-019-0573-z)
Supplement: Supplementary file 1 — Additional file 1: Table S1. Zero point energy (ZPE, a.u.), total energy (ET, a.u.), standard Gibbs free energy (Gθ, a.u.) and enthalpy (Hθ, a.u.) at 298.15 K obtained at the B3LYP/6-311++G**//LanL2DZ level for ten complexes. [file 13065_2019_573_MOESM1_ESM.docx]

**Table S1** Zero point energy (*ZPE*, a.u.), total energy (*E*_T_, a.u.), standard Gibbs free energy (*G*^θ^, a.u.) and enthalpy (*H*^θ^, a.u..) at 298.15 K obtained at the B3LYP/6-311++G**//LanL2DZ level for ten complexes

| **Complexes** | ***ZPE/*(a.u.)** | ***E_t_/*(a.u.)** | ***H*^θ^/( a.u.)** | **G^θ^/( a.u.)** |
| --- | --- | --- | --- | --- |
| **6MP-7** |  |  |  |  |
| **C1** | 0.102181 | -1976.634199 | -1976.607339 | -1976.706513 |
| **C2** | 0.101976 | -1976.639629 | -1976.612919 | -1976.709802 |
| **C3** | 0.102320 | -1976.635845 | -1976.609037 | -1976.707747 |
| **C4** | 0.102460 | -1976.637810 | -1976.611201 | -1976.706009 |
| **C5** | 0.101941 | -1976.641063 | -1976.614362 | -1976.710477 |
| **C6** | 0.102232 | -1976.636597 | -1976.610704 | -1976.704732 |
| **6MP-9** |  |  |  |  |
| **C7** | 0.101895 | -1976.628722 | -1976.601918 | -1976.699932 |
| **C8** | 0.102202 | -1976.635215 | -1976.608377 | -1976.706875 |
| **C9** | 0.101911 | -1976.630544 | -1976.603789 | -1976.700541 |
| **C10** | 0.102097 | -1976.634849 | -1976.607971 | -1976.705863 |
